# Inhibition of Influenza M2-Induced Cell Death Alleviates Its Negative Contribution to Vaccination Efficiency

**DOI:** 10.1371/journal.pone.0001417

**Published:** 2008-01-16

**Authors:** Petr O. Ilyinskii, Alexandra S. Gambaryan, Anatoli B. Meriin, Vladimir Gabai, Alex Kartashov, Galini Thoidis, Alexander M. Shneider

**Affiliations:** 1 Cure Lab, Canton, Massachusetts, United States of America; 2 Chumakov Institute of Poliomyelitis and Viral Encephalitis, Moscow, Russia; 3 Boston University School of Medicine, Boston, Massachusetts, United States of America; 4 Policy Analysis, Brookline, Massachusetts, United States of America; Ordway Research Institute, United States of America

## Abstract

The effectiveness of recombinant vaccines encoding full-length M2 protein of influenza virus or its ectodomain (M2e) have previously been tested in a number of models with varying degrees of success. Recently, we reported a strong cytotoxic effect exhibited by M2 on mammalian cells *in vitro*. Here we demonstrated a decrease in protection when M2 was added to a DNA vaccination regimen that included influenza NP. Furthermore, we have constructed several fusion proteins of conserved genes of influenza virus and tested their expression *in vitro* and protective potential *in vivo*. The four-partite NP-M1-M2-NS1 fusion antigen that has M2 sequence engineered in the middle part of the composite protein was shown to not be cytotoxic *in vitro*. A three-partite fusion protein (consisting of NP, M1 and NS1) was expressed much more efficiently than the four-partite protein. Both of these constructs provided statistically significant protection upon DNA vaccination, with construct NP-M1-M2-NS1 being the most effective. We conclude that incorporation of M2 into a vaccination regimen may be beneficial only when its apparent cytotoxicity-linked negative effects are neutralized. The possible significance of this data for influenza vaccination regimens and preparations is discussed.

## Introduction

M2 of influenza A virus is a 97-amino acid ion channel protein. M2 forms tetramers and is expressed at high density in the plasma membrane of infected cells. M2 transmembrane domain is highly conserved for all human, swine, equine, and avian strains of influenza A virus and is primarily responsible for proton translocation. This region is considered to be a target for the antiviral drugs, amantidine and rimantadine, that have been used for influenza prophylaxis and treatment over several decades [1].

M2 residues 1–24 comprise the extracellular domain (M2e), which is a target for antibody-mediated immunity. M2e is also extremely conserved. Since monoclonal antibodies to M2e were shown to provide protection against influenza-induced disease [2], various approaches utilizing M2e as part of a vaccine regimen have been evaluated as possible components of a broad-spectrum anti-influenza vaccine. Several reports presented evidence of high protection after DNA immunization with high doses of M2-encoding plasmid [3], [4], with one group reporting immunization with a combination of influenza nucleoprotein (NP) and M2 as the most efficient [4]. However, it is also known that excessive immunization with M2 fails to produce proportionate protection [5]. Moreover, when M2e domain was fused to NP, immunization of swine with such a DNA construct actually exacerbated the disease [6].

We recently reported that expression of influenza A virus M2 protein is highly toxic for mammalian cells *in vitro* and that its transmembrane region is essential for this function [7]. While similar observations have been made in yeast and insect cells, it was assumed that in heterotypic vertebrate expression systems, M2 causes no overt toxicity [8]. Contrary to that, we detected a significant M2-driven cytotoxic effect in 293 HEK cells, which was clearly linked to the proton-channeling ability of M2, with notable changes in mitochondria of pM2-transfected cells observed within the same time-period.

We hypothesized that M2-induced cytotoxicity may contribute negatively to the efficiency of recombinant and/or attenuated vaccines and that this is a molecular mechanism of the effect often called “insufficient M2 immunogenicity”. Indeed, we observed that adding a plasmid encoding a full-size M2 to the NP-based DNA vaccination regimen had a negative effect on animal survival following high-dose viral challenge. Furthermore, we observed that DNA vaccination with a multi-gene fusion construct (NP-M1-M2-NS1) that contains full size M2 and is not cytotoxic *in vitro* had a protective benefit that exceeds that of the construct that lacks M2 (NP-M1-NS1).

## Materials and Methods

### Plasmids and cells

The construction of NP and M2-containing plasmids (pNP and pM2) has been described earlier [7], [9]. Construction of plasmids encoding multi-gene fusion proteins NP-M1-NS1 (pNPM1NS1) and NP-M1-M2-NS1 (pNPM1M2NS1) was done by PCR-amplification from earlier described NP-, M1, NS1- and M2-expressing plasmids [7], [9]. Viral sequences were as follows: NP from strain A/WSN/33-H1N1, which is identical to A/PR/8/34-H1N1 on the amino acid level [9], M1 from the same strain, NS1 from strain A/PR/8/34-H1N1 and M2 from influenza A/WSN/33 (H1N1) strain (GenBank accession numbers: V01084, L25818, J02150 and L25818, correspondingly). Multi-gene sequences were first inserted into the pcDNA vector (Invitrogen, Carlsbad, CA, USA). HA-tag-encoding sequences were added at the 3′-termini and Flag-tag-encoding sequences were attached to 5′-termini of NP-M1-NS1 and NP-M1-NS1-M2 genes to enable their efficient immunological detection. All sequences were then cloned into the pCAGGS expression vector and used for expression testing and immunization [10].

### Transfection

293 HEK cells were transfected at 60–80% confluency in 35 mm plates with Lipofectamine 2000 (Invitrogen, Carlsbad, CA, USA) overnight (1.5 µg of total plasmid DNA per 5 µl LF2000). EGFP-expressing plasmid (0.5 µg) was used for co-transfection with pNPM1NS1 and pNPM1M2NS1 to visualize transfected cells (transfection efficiency was 80–90%). Control cells were transfected with the same amount of empty vector pCAGGS.

### Western blotting

Cells were lysed at 24 hours after transfection, normalized for protein concentration, and following SDS-PAGE and immunoblotting, NPM1NS1 and NPM1M2NS1 expression was detected using either anti-HA-tag or anti-Flag antibodies (Cell Signaling, Beverly, MA, USA).

### Cytotoxicity

In transfected cells was measured as a function of loss of GFP fluorescence as previously described [7]. Quantification of cell death was made by propidium iodide (PI) staining (5 µg/ml, 10 min). Images were taken at 16–90 hours after transfection under a fluorescent microscope (10× or 40× objective).

### Immunization with pNP, pM2, pNPM1NS1 and pNPM1M2NS1 in vivo

In the first experiment 5 µg of pNP, pM2 or pCAGGS (control) in 100 µl of PBS was injected intramuscularly per mouse per vaccination. Since the group immunized with a combination of pNP and pM2 received 10 µg of DNA total, the amount of DNA in other experimental groups was adjusted correspondingly with pCAGGS plasmid. Therefore, mice in the pNP group received 5 µg of pNP and 5 µg of pGACCS, mice in the pM2 group received 5 µg of pM2 and 5 µg of pGACCS, mice in the vector-immunized group received 10 µg of pGACCS. The size of the experimental groups was 20–22 animals per group with the exception of the control group of intact mice that comprised 10 animals. Mice were subjected to immunization with plasmid DNA three times with 14 days interval in between. Animal survival, weights and virus titers were monitored. For immunization with multi-gene fusion plasmids pNPM1NS1 and pNPM1M2NS1 25 µg of each plasmid was used. Mice (9–10 per group) were subjected to immunization with plasmid DNA three times with 14 days interval in between.

### Mouse-adapted influenza virus and animal infection

Avian influenza virus A/Mallard/Pennsylvania/10218/84 (H5N2) was obtained from the virus depository of the Virology Department of St. Jude Children's Research Hospital (Memphis, TN, USA) and was adapted to mice by lung-to-lung passage [11], [12]. Virus was propagated in 10-day-old embryonated chicken eggs. The virus-containing allantoic fluid was stored at −70°C and titrated in chicken embryo or in MDCK cells. Ether anaesthetized BALB/c mice (10–12 g) were infected intranasally with 50 µl of PBS-diluted allantoic fluid containing 5, 10 or 100 LD_50_ of A/Mallard/Pennsylvania/10218/84, 7 days after the last boost. Protection was measured by monitoring animal survival and body weight, which was assessed throughout an observation period of 21 days. Severely affected mice were euthanized. 1 LD_50_ of A/Mallard/Pennsylvania/10218/84 is equal to 100–1000 TCID_50_. Lung tissues from infected animals (2 from each group) were taken at day 4 after infection for viral titer evaluation. Viral titers were measured by focus assay in MDCK cells that were grown in 24-well plates and incubated with 0.5 ml/well of 10-fold sample dilutions. After 60-min absorption at RT, the virus inoculum was removed, cells washed and covered with 1% agarose. 50 hrs later, cells were fixed and incubated for 1 h with anti-influenza virus antibodies and visualized using peroxidase staining. Stained foci (PFU) were counted and titers calculated by the routine Reed & Muench method.

### Statistical methods

Standard error (SE) of a percent value was determined by the formula: SE = √p(100−p)/n, where p is percent value and n is number of animals used, similarly to that previously described [11]. Significance between two percent values (with probability 0.95): t = p_1_−p_2_/√SE_1_^2^+SE_2_^2^>2.0. Animal survival was compared using log-rank test (PROC LIFETEST, SAS(R) statistical package). The differences at P-value below 0.05 were considered significant.

## Results

### Inclusion of M2-encoding plasmid into vaccination regimen may result in disease exacerbation

**Figure 1:**
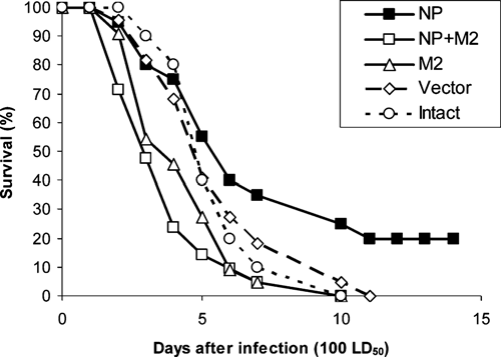
Survival of mice vaccinated with the combinations of pNP and pM2 after challenge with 100 LD50 of H5N2 influenza virus strain A/Mallard/Pennsylvania/10218/84. Animals were immunized and challenged as described in Materials and Methods.

The level of protection provided by DNA vaccination with pNP, pM2, pNP+pM2 or vector plasmid was assessed for animal groups challenged with 100 LD_50_ of mouse-adapted H5N2 influenza virus. Vaccination with pNP resulted in 20% survival (Fig. 1). Protection data unequivocally suggested that in such experimental settings inclusion of pM2 into the immunization regimen is detrimental for protection (Fig. 1). Specifically, while the pM2-immunized group showed no effect on the dynamics of survival and disease similar to vector-immunized controls, the survival in pNP+pM2-immunized group was clearly impaired compared to immunization with pNP alone (Fig. 1) and this difference was statistically significant (p<0.05). There was no difference in viral titers between pNP and pNP+pM2 immunized groups, with the titer in the pM2-immunized group being somewhat lower (not shown).

### Expression of fusion proteins derived from conserved influenza genes

**Figure 2:**
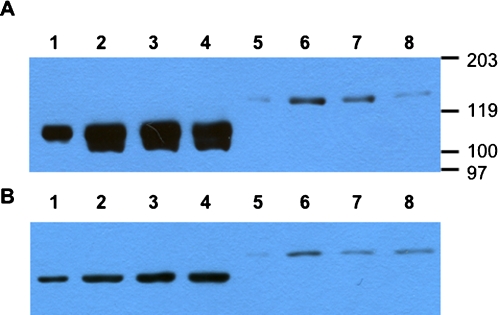
Expression of recombinant multi-partite fusion proteins based on conserved genes of influenza in vitro. Lanes 1–4 - cells transfected with pNPM1NS1, lanes 5–8 - with pNPM1M2NS1. A - anti-Flag-tag antibodies used for protein detection; B - anti-HA-tag antibodies used. Lanes 1, 2, 5 and 6 - starting point of chase (0 hours). Chase time: 3.5 hours (panel A, lanes 3 and 7), 7 hours (panel A, lanes 4 and 8), 8 hours (panel B, lanes 3, 4, 7 and 8). Proteosome inhibitor MG132 added to samples in panel B, lanes 4 and 8. Lanes 1 and 5 contain ⅓ of total protein loaded to lanes 2–4 and 6–8, correspondingly. Position of molecular weight markers is shown.

**Figure 3:**
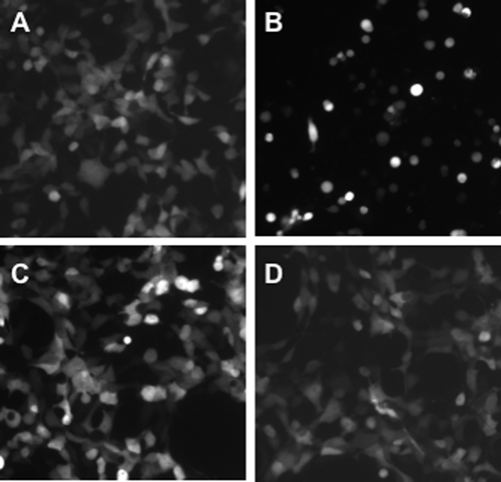
Absence of cytotoxicity induced by multi-partite fusion proteins based on conserved genes of influenza containing and not containing M2 sequence (as measured by green fluorescence). HEK cells were co-transfected with 0.2 µg of pGFP and 0.8 µg of the following plasmids: A - pCAGGS, B - pM2, C - pNPM1NS1, D - pNPM1M2NS1. Images were taken 64 hours after transfection.

Plasmids encoding fusion genes, pNPM1NS1 and pNPM1M2NS1, that contained full sequences of conserved influenza NP, M1 and NS1 genes (with or without M2) were constructed and shown to express the proteins of predicted size (Fig. 2). They were tested for their expression *in vitro* using antibodies to N-terminal Flag-tag (Fig. 2A) or C-terminal HA-tag (Fig. 2B). It was clear that inclusion of M2-encoding sequence resulted in dramatic impairment of expression of the fusion proteins, with NPM1NS1 construct expressing at least 10 times more efficiently than NPM1M2NS1 (Fig. 2A). Higher levels of expression of the three-partite over four-partite fusion protein was also seen when antibody to C-terminal HA-tag was used (Fig. 2B), although the overall difference was less. Neither of the fusion proteins exhibited any cytotoxicity similar to that induced by wild-type M2 (Fig. 3). Specifically, for NPM1M2NS1 no signs of cytotoxicity were seen up to 96 hours after transfection even when its efficiency of transfection was 90–100% (not shown).

### Protection of mice from disease by immunization with plasmids encoding fusion proteins of influenza virus

**Figure 4:**
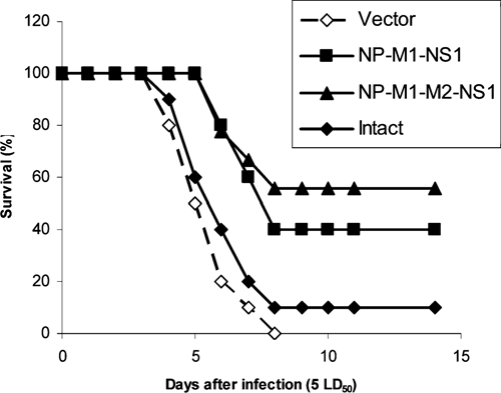
Survival of mice vaccinated with pNPM1NS1 and pNPM1M2NS1 after challenge with 5 LD50 of H5N2 influenza virus strain A/Mallard/Pennsylvania/10218/84. Animals were immunized and challenged as described in Materials and Methods.

We utilized both multi-partite fusion constructs in vaccination experiments in order to test whether the utilization of M2 in a fashion that alleviates its cytotoxicity is capable of providing additional protective benefit (Fig. 4). Weakly expressed NPM1M2NS1 construct resulted in partial protection against 5 LD_50_ of mouse-adapted H5N2 virus, which was statistically significant (p<0.05) and exceeded that exhibited by the much more efficiently expressed NPM1NS1 construct which did not include M2, although the latter also provided statistically significant protection as shown by log-rank analysis.

## Discussion

The usefulness of the immune response against M2 for protection against influenza-induced disease has been known for a long time and multiple attempts have been undertaken to exploit this for the generation of cross-protective vaccine preparations [3], [4], [13], [14], [15]. Despite significant advances along this route, this aim has not been accomplished. Moreover, in several experimental settings, the results have been disappointing [6]. Our results presented herein (Fig. 1) coupled with earlier accumulated data [6] clearly indicate that immunization with M2 may lead to disease exacerbation.

Our data point to the possibility that M2-dependent cytotoxicity is a factor in influenza pathogenesis, and also raises the question whether any vaccine (recombinant, live-attenuated or to a lesser extent inactivated) containing full-size functional M2 may be detrimental or even harmful. The molecular mechanism of this negative action of M2 may be, at least in part, linked to its cytotoxic effect which we have recently described [7]. If M2-induced cytotoxicity is linked to suppression of the immune response in M2-containing vaccines, then the incorporation of influenza M2 into a vaccination regimen should include the neutralization of its possible negative effects.

There are several approaches on how to neutralize M2 cytotoxic activity. In particular, it is possible to incorporate M2 into a multi-gene fusion construct, designed to limit the accessibility of M2 functional domains. We have recently demonstrated that addition of M1 and NS1 to NP is advantageous in several animal models of DNA vaccination [9]. In this study, we tested a fusion gene including these conserved influenza genes with or without M2, with the sequence of the latter engineered to be inside and not on the termini of the fused gene (resulting in NPM1M2NS1 construct).

The expression level of this NPM1M2NS1 fusion protein was at least 10 times lower than that of NPM1NS1 (Fig. 2). At the same time, the incorporation of M2 sequence into the third position of the recombinant molecule did not result in any overt cytotoxicity *in vitro*, different from full-size M2 (Fig. 3). Additionally, the four-partite NPM1M2NS1 fusion protein was more protective than NPM1NS1 protein in a DNA vaccination model, while expressed at significantly lower levels (Figs. 3, 4). It is plausible to suggest that some of the detrimental effects exhibited by M2 are alleviated when it is placed in the central position of the recombinant protein and therefore the resulting protein is not cytotoxic.

Collectively, it is reasonable to suggest that expression of wt-M2 protein, although useful as an antigen, also contributes negatively to the vaccination regimen by virtue of its cytotoxic capacity. Therefore, successful utilization of M2 in anti-influenza immunization may require elimination of its cytotoxicity without interfering with its antigenic epitopes. This may have been partially accomplished in approaches that employ M2e domain [14], [15]. Incorporation of full M2 sequence into multi-gene recombinant structures based on conserved influenza proteins is yet another avenue to attain this goal as was accomplished with the NPM1M2NS1 fusion construct described herein. This may in turn be complemented by M2 site-specific mutagenesis. We have previously reported that introducing hydrophobic amino acids within the ion channel formed by M2 tetramer is an effective strategy to reduce M2 cytotoxicity [7].
